# Supplementary material for: Organizational Principles of the Centrifugal Projections to the Olfactory Bulb
Source: Int J Mol Sci. 2023 Feb 26;24(5):4579. doi: 10.3390/ijms24054579 (PMC10002860; doi:10.3390/ijms24054579)
Supplement: Supplementary file 1 [file ijms-24-04579-s001.zip › ijms-2151657-supplementary.pdf]

Supplemental Information

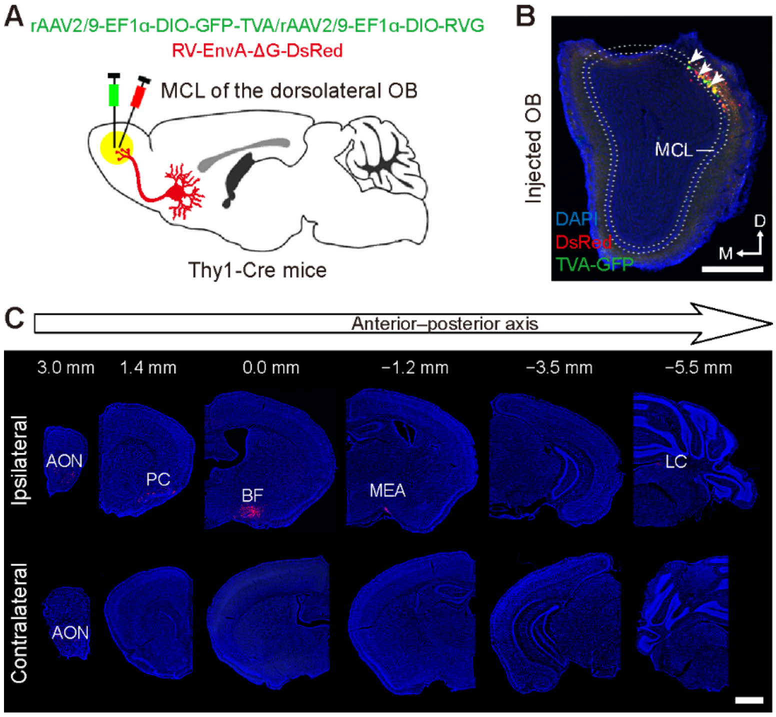

Supplementary Figure S1. Retrograde tracing of monosynaptic input to the M/TCs of the dorsolateral OB. (**A**) Strategy for input tracing from the M/TCs using RV-based retrograde mono-synaptic tracing viruses. (**B**) Example image showing starter neurons (indicated by arrows) in the MCL of the dorsolateral OB. MCL, mitral cell layer. (**C**) Example images showing fluorescently labeled input neurons in major brain areas. AON, anterior olfactory nucleus; PC, piriform cortex; BF, basal forebrain; MEA, medial amygdalar nucleus; LC, locus coeruleus. Scale bars, 500 μm in (**B**); 1 mm in (**C**).

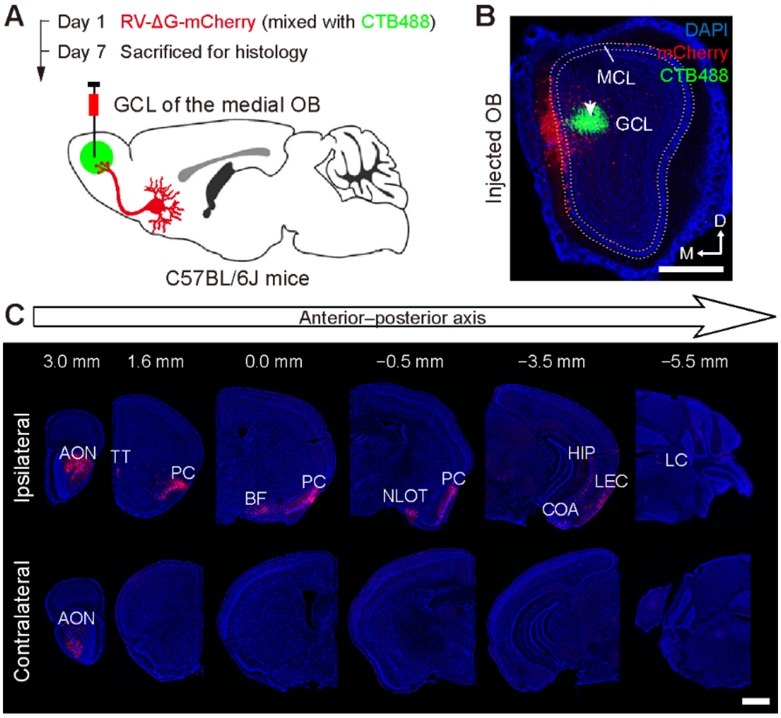

Supplementary Figure S2. Retrograde tracing of input to the GCs of the medial OB. (**A**) Strategy for input tracing from the GCs using RV-ΔG-mCherry. (**B**) Example image showing the injection site (indicated by arrow) in the GCL of the medial OB. (**C**) Example images showing fluorescently labeled input neurons in major brain areas. AON, anterior olfactory nucleus; PC, piriform cortex; TT, taenia tecta; BF, basal forebrain; NLOT, lateral olfactory tract nucleus; HIP, hippocampal region; LEC, lateral entorhinal cortex; COA, cortical amygdalar nucleus; LC, locus coeruleus. Scale bars, 500 μm in (**B**); 1 mm in (**C**).
